# Reduced cortical thickness in right Heschl’s gyrus associated with auditory verbal hallucinations severity in first-episode schizophrenia

**DOI:** 10.1186/s12888-015-0546-2

**Published:** 2015-07-07

**Authors:** Xudong Chen, Shengxiang Liang, Weidan Pu, Yinnan Song, Tumbwene E. Mwansisya, Qing Yang, Haihong Liu, Zhening Liu, Baoci Shan, Zhimin Xue

**Affiliations:** 1 Mental Health Institute of the Second Xiangya Hospital, Key Laboratory of Psychiatry and Mental Health of Hunan Province, Central South University, Changsha, Hunan 410011 People’s Republic of China; 2 Key Laboratory of Nuclear Analysis Techniques, Institute of High Energy Physics, Chinese Academy of Sciences, Beijing, People’s Republic of China; 3 Medical Psychological Institute of the Second Xiangya Hospital, Central South University, Changsha, Hunan People’s Republic of China; 4 Department of Clinical Nursing and Community Health, the University of Dodoma, Dodoma, Tanzania; 5 Department of Medicine, Yale-New Haven Hospital, Yale School of Medicine, New Haven, CT USA; 6 Mental Health Center of Xiangya Hospital, Central South University, Changsha, Hunan People’s Republic of China

**Keywords:** Schizophrenia, Auditory hallucination, Cortical thickness, Magnetic resonance imaging, Heschl’s gyrus

## Abstract

**Background:**

Auditory verbal hallucinations (AVHs) represent one of the most intriguing phenomena in schizophrenia, however, brain abnormalities underlying AVHs remain unclear. The present study examined the association between cortical thickness and AVHs in first-episode schizophrenia.

**Method:**

High-resolution MR images were obtained in 49 first-episode schizophrenia (FES) patients and 50 well-matched healthy controls (HCs). Among the FES patients, 18 suffered persistent AVHs (“auditory hallucination” AH group), and 31 never experienced AVHs (“no hallucination” NH group). The severity of AVHs was rated by the Auditory Hallucinations Rating Scale (AHRS). Cortical thickness differences among the three groups and their association with AVHs severity were examined.

**Results:**

Compared to both HCs and NH patients, AH patients showed lower cortical thickness in the right Heschl’s gyrus. The degree of reduction in the cortical thickness was correlated with AVH severity in the AH patients.

**Conclusions:**

Abnormalities of cortical thickness in the Heschl’s gyrus may be a physiological factor underlying auditory verbal hallucinations in schizophrenia.

**Electronic supplementary material:**

The online version of this article (doi:10.1186/s12888-015-0546-2) contains supplementary material, which is available to authorized users.

## Background

Schizophrenia is one of the most debilitating mental illnesses [1], characterized by positive symptoms, negative symptoms and cognitive deficits. As a core positive symptom, auditory verbal hallucinations (AVHs) is an important diagnostic criterion for schizophrenia [2], which has been shown to be closely related to schizophrenia patients’ long-term social functioning and quality of life [3].

Previous neuroimaging studies have revealed widespread morphological abnormalities associated with AVHs, such as gray matter loss in the frontal [4, 5], insular [6], and anterior/posterior cingulate [7, 8] cortices. The most extensively focused brain region in the investigation of AVHs is the temporal lobe, in particular the primary auditory cortex (PAC, also known as Heschl’s gyrus), and the secondary auditory cortex [9]. Both are known to be crucial for auditory perception and comprehension [10]. Several lines of evidence have shown that schizophrenia patients with AVHs present reduced gray matter volumes in the superior and transverse temporal gyri [11–13]. Furthermore, a recent meta-analysis of voxel-based morphometry (VBM) studies included a large sample totaling 438 patients (307 with AVHs) and confirmed a significant correlation between the extent of gray matter loss in the Heschl’s gyrus (HG) and the severity of AVHs [14]. Interestingly, by applying a manual segmentation method, another study found that hallucinating patients exhibited increased volume in the HG [15]. Yet another study found that schizophrenia patients with AVHs had increased volumes of temporal gray and white matter compared patients without AVHs [4]. The inconsistent findings may be accounted for by differences in methodology [14], hallucination history [16], symptom diversity [17], substance abuse [18], illness course [19, 20], and antipsychotic medication use [21]. Thus, controlling potentially confounding factors listed above may help provide more reliable evidence on the alternations in brain structures underpinning AVHs.

In the present study, we recruited two groups of first-episode schizophrenia patients, who were carefully matched for socio-demographic variables, illness course, medication dose, and symptom severity (except for the AVHs). The auditory hallucination (AH) group included patient with current and persistent AVHs. Patients without current hallucinations or with only occasional hallucinations based on the Scale for Assessment of Positive Symptoms (SAPS) were excluded. In the no hallucination (NH) group, patients with any AVH history were excluded. (For detailed inclusion and exclusion criteria, please see Methods). Furthermore, a group of healthy controls (HCs) was also recruited, matching the patients in age, sex and years of education.

By applying a novel surface-based analysis approach to the matched schizophrenia patient samples, we aimed at characterizing the cortical thickness in the first-episode schizophrenia patients with and without AVHs. We expected patients with AVHs to show altered cortical thickness correlating with their AVH severity.

## Methods

### Study subjects

Forty-nine right-handed first-episode schizophrenia (FES) patients who were diagnosed with schizophrenia within the past 18 months with no previous episodes of psychosis as per Diagnostic and Statistical Manual for Mental Disorders Fourth Edition (DSM-IV), determined by the Structured Clinical Interview for DSM-IV Axis I Disorders, Patient Edition (SCID-I/P) [22], were recruited from an inpatient unit at the Second Xiangya Hospital of Central South University, Changsha. Inclusion criteria for both patients and healthy controls were similar: (1) age between 18 and 45 years old; (2) Han Chinese; and (3) sufficient understanding and expressive capacity with ≥9 years of education. Exclusion criteria for participants were: (1) moderate to severe learning disability; (2) a history of substance abuse; (3) a history of brain trauma or neurological disease; (4) left-handedness; and (5) previous electroconvulsive therapy and any other contraindications to MRI. Diagnosis of schizophrenia and the symptom ratings were determined by the consensus of two experienced psychiatrists (Z.L. and H.L.). The hallucination history was determined based on the SCID-I/P hallucinations items. Of the patients with first-episode schizophrenia, 18 suffered persistent AVHs and were classified as the auditory verbal hallucinations group (AH). In particular, patients were assigned to the AH group if they scored >2 on item 2 or 3 of the SAPS and patients with occasional hallucinations were excluded (hallucinations score = 2). The other 31 patients with first-episode schizophrenia had never experienced auditory verbal hallucinations (no hallucinations group, NH).

Fifty right-handed healthy controls (HCs) were recruited from the city of Changsha through poster advertisements and screened. HCs were evaluated using the SCID-Non-Patient Edition to confirm the lifetime absence of psychiatric and neurological illnesses [23]. HCs who met the DSM-IV criteria for an Axis-I psychiatric disorder were excluded. Additionally, healthy subjects were interviewed to confirm that there was no history of psychiatric illnesses among their first-degree relatives. The Ethics Committee at Second Xiangya Hospital of Central South University reviewed and approved the study. All participants received a description of the experimental procedures and gave written informed consent to participate in the study.

Participants’ IQ was estimated using the information subscale and digit-symbol coding subscale of the Wechsler Adult Intelligence Scale (WAIS)-Chinese version [24]. In subjects with schizophrenia, positive and negative symptoms within 1 month prior to the study were assessed using the Scale for Assessment of Positive Symptoms (SAPS) [25] and the Scale for the Assessment of Negative Symptoms (SANS) [26], respectively. AVH severity was evaluated using the Auditory Hallucinations Rating Scale (AHRS). This questionnaire assesses multiple characteristics of AVHs, such as the frequency of occurrence, length of episodes, loudness of the voices, and influence and discomfort of AVHs as experienced by the patient [27]. Dosage of antipsychotic medication equivalent to 100 mg/day of chlorpromazine (CPZ) was also calculated [28]. Handedness was ascertained using the Annett Hand Preference Questionnaire [29].

To minimize the effects of chronicity of illness and neuroleptic medications on brain structure, this study preferentially recruited patients who were just beginning treatment with antipsychotic medication for the first time and matched the medication dose between the two patient groups. Current and previous antipsychotic regimens (medication and duration of use) were recorded. In the patient groups, two patients (4 %) never received antipsychotic treatment, 43 patients had been on antipsychotic treatment for less than 4 weeks and four patients had been treated for more than 4 weeks. One patient (2 %) was receiving first-generation antipsychotics (FGAs) monotherapy (sulpiride, 1 case); 41 patients (84 %) were receiving second-generation antipsychotics (SGAs) monotherapy (clozapine, 1 case; risperidone, 19 cases; quetiapine, 2 cases; olanzapine, 15 cases; aripiprazole, 1 case; ziprasidone, 3 cases) and five patients (10 %) were receiving combination antipsychotic therapy (combining an SGA and an SGA). Mean current treatment duration was 11.92 (SD = 13.52) days and mean lifetime exposure to antipsychotics was 13.06 (SD = 14.95) days among the 49 patients.

### Magnetic resonance imaging acquisition

High-resolution T1-weighted images were acquired using T1-weighted 3D turbo field echo (T1W-3D-TFE) on a 3.0-Tesla Philips Achieva MRI scanner (Philips, The Netherlands) with the following parameters: repetition time = 7.5 ms, echo time = 3.7 ms, flip angle = 8°, field of view = 240 × 240 mm^2^, acquisition matrix = 256 × 200, slice thickness = 1 mm, gap = 0, number of slices = 180. Foam pads and earplugs were used to minimize head motion and scanner noise. All scans were inspected for motion artifacts. The absence of gross pathological findings was confirmed by a neuroradiologist. All patients and controls were examined during the same study period and there was no scanner upgrade during this time.

### Cortical thickness measurement and cortical parcellation

Images were processed and cortical thickness was measured using the longitudinal stream in FreeSurfer version 5.2.0 (http://surfer.nmr.mgh.harvard.edu/fswiki/recon-all/), which has been validated in previous research [30]. All the processing steps, including motion correction, skull stripping, registration to Talairach space, intensity normalization, segmentation of the white matter and gray matter, tessellation of the gray-white boundary, automatic topology correction and surface deformation, were completed using the fully automated pipeline without any manual intervention. Cortical thickness was calculated as the average of the closet distance from the white matter to the vertex and the closet distance from the vertex to the white matter [31]. The cortex of each hemisphere was automatically parcellated into 34 regions (based on [32], available from http://surfer.nmr.mgh.harvard.edu/fswiki/CorticalParcellation), subdividing the human cerebral cortex into standard gyral neuroanatomical regions. This highly reliable parcellation method has been adopted in a line of previous studies [33, 34]. The average cortical thickness of each region from both FES patients and HCs were extracted. All results of cortical parcellations were examined by both visual inspection and FreeSurfer QA Tools (available from http://www.freesurfer.net/fswiki/QATools).

#### Statistics

Statistical analysis was performed with Statistical Package for Social Sciences, version 17.0 (SPSS Inc., Chicago, Illinois). Differences in age and educational levels among the three groups were assessed using Analyses of Variance (ANOVA). Gender differences were compared using the chi-squared test. The average cortical thickness in the 34 regions of each hemisphere was extracted and entered into SPSS software using a general linear model and compared among the patient groups and healthy controls, with age and sex serving as covariates. Significance was set to *p* < .05, corrected for multiple comparisons using Least Significant Difference (LSD).

Pearson correlation analysis was performed on the average cortical thickness in regions with altered thickness and the AHRS scores, and the total score of the SAPS and SANS to examine potential associations between anatomical deficits and clinical symptoms. Furthermore, we used Pearson correlation to quantify the relationship between the duration of illness and the alterations in cortical thickness. *P* < .05 was considered significant. Additionally, given that the CPZ equivalent of current medication dose did not follow a normal distribution, Spearman's rank correlation analysis between cortical thickness and medication dose was performed.

## Results

### Demographic and clinical characteristics

Demographic and clinical characteristics of the groups are shown in Table 1. There were no significant differences among the groups regarding age, sex, and years of education. No difference emerged with respect to the duration of illness or medication dosage between the AH and NH patient groups. Relative to the NH patients, the AH patients scored significantly higher on total SAPS score, but not for total SAPS score minus hallucinations subscales or total SANS score. In addition, there was no significant difference between the two patient groups in any of the sub-scores of SANS or SAPS except for hallucinations (see Table 1).

**Table 1:** Demographic and clinical characteristics of participants^a^

	AH	NH	HC	Chi-Square Analysis/t tests		
	(*n* = 18)	(*n* = 31)	(*n* = 50)	*F/χ2/t*	*p*	Post Hoc
Demographics						
Age, years^b^	24.11(6.27)	24.29(5.92)	24.98(6.60)	*F* = .18	.835	
Sex,n(% male)	12(66.67 %)	17(54.80 %)	25(50.00 %)	*χ2* = 1.48	.479	
Education, years^c^	12.42(2.92)	12.50(2.22)	13.2(2.16)	*F* = 1.22	.299	
Duration of illness,months^d^	6.33(4.78)	7.09(4.48)		t = −.56	.579	
WAIS-Information	17.89(6.07)	16.31 (4.42)	20.22(4.50)	*F* = −3.32	.002*	NH < HC
WAIS-Digit symbol	66.00(16.97)	68.00(15.61)	86.56(16.01)	*F* = −6.14	<.001*	AH, NH < HC
Clinical Variables						
AHRS	27.06(6.32)					
SAPS						
Global rating of Hallucinations	2.78(1.93)	.45(1.12)		*t* = 5.36	<.001*	
Global rating of Delusions	2.27(1.45)	2.81(1.80)		*t* = −1.06	.293	
Global rating of BizarreBehaviour	.56(1.20)	.87(1.15)		*t* = −.91	.366	
Global rating of Positive Formal Thought Disorder	.83(1.25)	.68(1.17)		*t* = .44	.662	
Total	27.78(14.65)	16.67(11.52)		*t* = 2.94	.005*	
Total – Hallucinations	14.89(10.82)	15.58(11.25)		*t = −*.21	0.834	
SANS						
Global Rating of Affective Flattening	1.06(1.21)	1.35(1.50)		*t* = −.72	.474	
Global Rating of Alogia	0.83(1.20)	1.26(1.44)		*t* = −1.06	.296	
Global Rating of Avolition - Apathy	1.28(1.23)	1.68(1.70)		*t* = −.87	.388	
Global Rating of Anhedonia- Asociality	1.67(1.33)	2.42(1.59)		*t* = −1.70	.097	
Global Rating of Attention	0.83(0.92)	1.10(1.40)		*t* = −.71	.480	
Total	21.44(19.92)	30.12(25.44)		t = −1.24	.220	
Chlorpromazine equivalents (mg)	189.35(62.09)	250.53(201.54)		*t* = −1.25	.218	

*p < .05

*HC* healthy controls, *AH* hallucinating patients with schizophrenia, *NH* non-hallucinating patients with schizophrenia, *Total-Hallucinations* total SAPS score minus hallucinations subscales score

*AHRS* Auditory Hallucinations Rating Scale, *SAPS* Scale for the Assessment of Positive Symptoms, *SANS* Scale for the Assessment of Negative Symptoms, *WASI* Wechsler Abbreviated Scale of Intelligence

^a^Mean and SD are reported unless otherwise specified. Analyses of demographics and clinical data wereperformed in SPSS (http://www.spss.com). All tests were two-tailed

^b^Age was defined as age at the time of magnetic resonance imaging scanning

^c^Years of education refers to the total number of years of completed education as reported by the participant

^d^Duration of illness was defined as number of years between age at onset and age at MRI scanning

### Alterations in patient cortical thickness

**Fig. 1:**
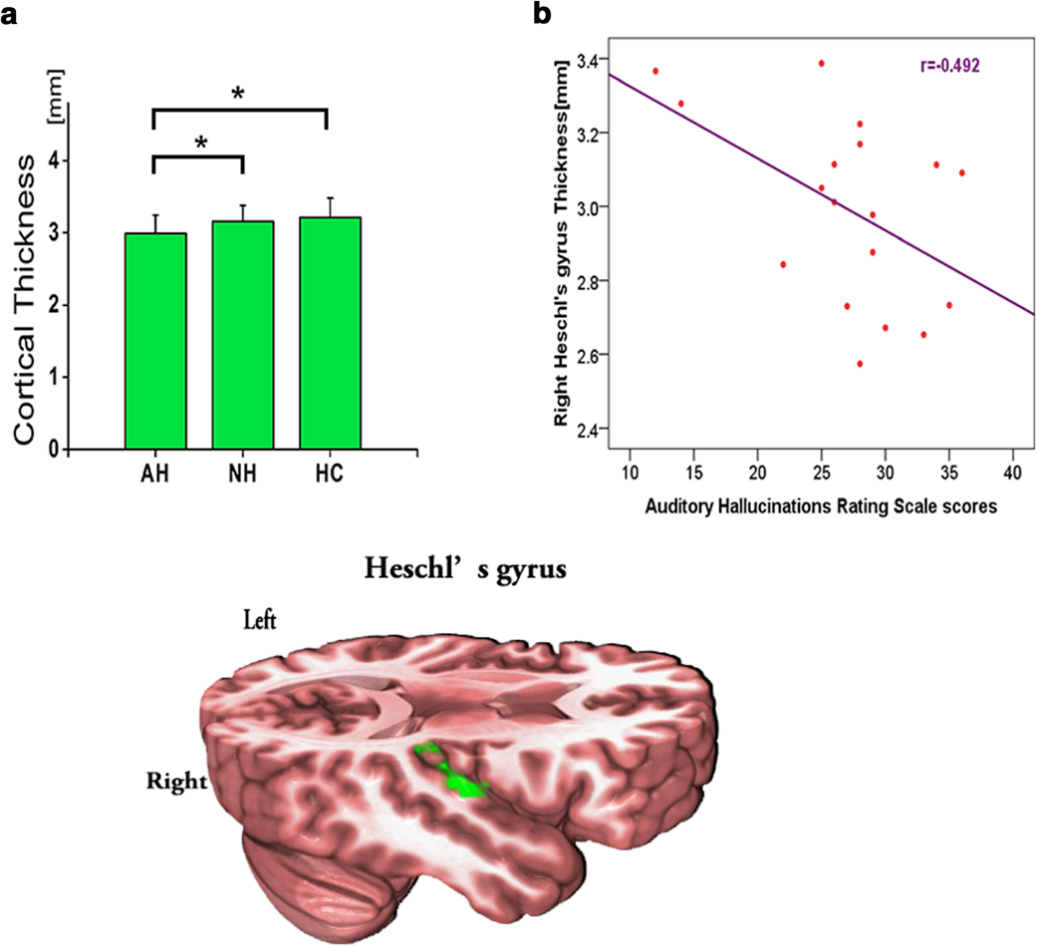
Cortical thickness analysis among patients and healthy controls groups. **a** Significant differences among groups were identified using ANCOVA with age and sex as covariates (* p < .05, corrected for multiple comparisons with LSD). The right Heschl’s gyrus (labeled in green) showed significant difference in cortical thickness among three groups. (AH < NH, HC, AH = Hallucinating patients with schizophrenia, NH = Non-hallucinating patients with schizophrenia, HC = Healthy controls. Error bars represent SDs). **b** The cortical thickness of right Heschl’s gyrus was negatively correlated with the severity of auditory verbal hallucinations, as assessed by AHRS scores

Group ANCOVA analysis demonstrated that only the right transverse temporal cortex (Heschl’s gyrus) showed significant difference in cortical thickness among the three groups (*F* = 6.06 *p* < .05; see Table 2 and Additional file 1: Table S1). Post-hoc comparisons showed that the right HG cortical thickness was significantly reduced in the AH patients compared to that of the NH patients as well as the HCs (*p* < .05, corrected for multiple comparisons with LSD; see Table 2, Fig. 1)

**Table 2:** Cortical thickness of schizophrenia patients and healthy controls in right Heschl’s gyrus

	Cortical thickness(mm)		ANCOVA		Pairwise comparisons^a^		
	Mean	SD	F	*P* values		Mean difference	*P* values
AH	2.99	.25			AH < NH	-.161	.021*
NH	3.16	.22	6.06	.003**	NH < HC	.062	.246
HC	3.21	.27			AH < HC	-.223	.001*

*p < .05, **p < .01

*HC* healthy controls, *AH* hallucinating patients with schizophrenia, *NH* non-hallucinating patients with schizophrenia

^a^Adjustment for multiple comparisons: Least Significant Difference

### Correlation between alterations in cortical thickness and clinical symptoms severity

In the AH group, the average cortical thickness of the region with reduced cortical thickness (*i.e.*, right HG) negatively related to the severity of AVHs, as assessed by AHRS scores, but not with SAPS or SANS (see Fig. 1). Greater reduction in the cortical thickness was observed in patients with more severe AVHs. No significant correlation was observed between the cortical thickness in the region with altered thickness and the duration of illness (*r* = −.22 *p* > .05), or the medication dosage in the patient groups (*r* = .14, *p* > .05).

## Discussion

In line with our hypothesis, we found that reduced cortical thickness in Heschl’s gyrus was significantly related to auditory hallucinations in FES patients. One advantage of the present study, which specifically isolated patients with current and persistent auditory hallucinations and compared them with matched non-hallucinating patients and healthy controls, is phenotypic homogeneity to explore the neural correlates of hallucinations. Notably, our patient groups differed only in terms of AVH symptomatology, minimizing the confounding effect of other clinical characteristics.

Several advantages of this study, particularly on the controlling confounding factors, are of strong interest for identifying the specific anatomical deficits associated with verbal hallucinations. First, excessive cortex thinning over time in the frontal and temporal areas has been demonstrated in schizophrenia and the changes can progresses across the entire course of the illness [35]. We recruited patients with first-episode schizophrenia with relatively short illness duration to minimize confounding effects of the chronic illness course on the neural substrate of hallucination. Second, association between psychotic symptoms and different deficits in cortical thickness has been observed [36]. Moreover, it has been indicated that antipsychotic drugs affect the regional cerebral volume, particularly in the frontal lobes [37, 38] and caudate nucleus [39]. Therefore, we matched the symptoms severity (other than hallucinations) and medication dosage between the two patient groups, further facilitating the identification of specific neuro-substrate of hallucinations. Finally, we used the surface-based cortical thickness approach, which can acquire data at the sub-voxel resolution [40], reflecting the size, density, and arrangement of neurons, glial cells, and synaptic spines [41]. Given that surface-based methods are designed to remove individual variability in local cortical folding [31], the cortical thickness might elucidate the exact nature of potential structural alterations in schizophrenia.

The Heschl’s gyrus belongs to the primary auditory cortex [42], which is surrounded by the secondary auditory processing areas on the superior temporal gyrus (STG). Our findings are consistent with a recent meta-analysis of voxel-based morphometry study [14], which revealed that the severity of AVHs was significantly associated with gray matter volume (GMV) reductions in bilateral HG and STGs. HG structural abnormality has been reported to relate with the severity of auditory hallucinations [13], which is also replicated in our study. In adolescent schizophrenia patients [43], gray and white matter volume reduction in the HG has been observed. Furthermore, cortical thinning in the HG was reported in both high-risk neonates and adults with schizophrenia [34, 44], suggesting that the HG structural abnormalities may relate to genetic predispositions.

Previous studies also reported structural abnormalities in other brain regions, such as frontal [4, 5], temporal [9] and insular cortex [6], whereas we did not observe any difference in cortical thickness in these areas. Such contradictory findings may be attributed to differences of methodology and schizophrenia heterogeneity. First, in contrast to our surface-based morphometry analysis, most previous studies applied the voxel-based morphometry (VBM) approach. The VBM approach mainly focuses on the structrual changes in the gray matter volume or density, not cortical thickness. To the best of our acknowledge, there has been only one previously published study assessing cortical thickness in a relatively small sample of 10 chronically hallucinating schizophrenic patients, which found cortical thickness increase in the parahippocampal gyrus and reduction in the frontal and temporal cortex [45]. Schizophrenia is a very heterogeneous mental disorder. Patients present with a variety of clinical symptoms and durations of illness. Different from that study, all patients in our study were in their first-episode (mean duration of illness 6.8 months). Evidence has demonstrated that schizophrenia patients show cortex thinning over time across the entire course of illness [35], suggesting that the chronicity of the illness might be an important contributor to cortical thinning. Moreover, in the present study, we did not observe significant thickness reduction in the left HG. This finding concurs with previous studies demonstrating that in the primary auditory cortex, the morphometric changes appeared to be more consistent on the right than the left in schizophrenia patients [46, 47].

Although the underlying neuropathology of the HG gray matter reduction is not clear, postmortem studies have suggested that cortical thinning may reflect a loss of neuropil or altered pruning [48, 49]. Based on the knowledge of previous studies and our results, the anatomical deficits in the primary auditory cortex may represent a core pathology of AVHs during the early course of schizophrenia.

This study has some limitations. First, the FES patients included in the study were not completely treatment-naïve; most of them had received a relatively short course of antipsychotic medications. Studies showed that long term antipsychotic medication has a significant impact on brain morphology [50]. However, the two patient groups here did not differ in terms of medication dosage or duration. In addition, when we further controlled for medication effects by performing correlation analysis with CPZ-equivalents in patient groups, and there was no significant correlation between CPZ-equivalent and cortical thickness. Second, our analysis method only allowed inference on cortical thickness alterations. Due to the limitations associated with the MRI structural measurements, the effects of vascularization and tissue fluid levels cannot be excluded. Third, although the total FES group is reasonably large, the power of the study might be limited by dividing patients into subgroups with and without AVHs. Given the cross-sectional nature of this study, we cannot exclude a potential impact of schizophrenia heterogeneity on our findings and future longitudinal studies are warranted to better understand the developmental context of these structural alterations in patients with auditory hallucinations.

## Conclusion

In conclusion, the present study demonstrated that first-episode schizophrenia patients with AVHs exhibit reduced cortical thickness in the right HG that was associated with severity of auditory hallucinations. This finding suggests that anatomical deficits in primary auditory cortex gyrus may modulate the pathogenesis of auditory verbal hallucinations in the early stage of schizophrenia.

## Additional file

Additional file 1: **Table S1.** Regional cortical thickness of schizophrenia patients and healthy controls.

## Abbreviations

AVHs: Auditory verbal hallucinations
HCs: Healthy controls
FES: First-episode schizophrenia
AHRS: Auditory Hallucinations Rating Scale
AH: Auditory hallucination
NH: No hallucination
PAC: Primary auditory cortex
VBM: Voxel-based morphometry
HG: Heschl’s gyrus
SAPS: Scale for Assessment of Positive Symptoms
SANS: Scale for the Assessment of Negative Symptoms
DSM-IV: Diagnostic and Statistical Manual for Mental Disorders Fourth Edition
SCID-I/P: Structured Clinical Interview for DSM-IV Axis I Disorders, Patient Edition
WAIS: Wechsler Adult Intelligence Scale
CPZ: Chlorpromazine
ANOVA: Analyses of Variance
SPSS: Statistical Package for Social Sciences
STG: Superior temporal gyrus
GMV: Gray matter volume
