# Supplementary material for: Reduced cortical thickness in right Heschl’s gyrus associated with auditory verbal hallucinations severity in first-episode schizophrenia
Source: BMC Psychiatry. 2015 Jul 7;15:152. doi: 10.1186/s12888-015-0546-2 (PMC4493802; doi:10.1186/s12888-015-0546-2)
Supplement: Additional file 1: — Table S1. Regional cortical thickness of schizophrenia patients and healthy controls. [file 12888_2015_546_MOESM1_ESM.doc]

**Table S1** Regional cortical thickness of schizophrenia patients and healthy controls ^a^

| Regions | AH | | NH | | HC | | ANCOVA | |
| --- | --- | --- | --- | --- | --- | --- | --- | --- |
|  | Estimated marginal mean | SE | Estimated marginal mean | SE | Estimated marginal mean | SE | F | P |
| **Frontal lobe** |  |  |  |  |  |  |  |  |
| L superior frontal | 3.65 | .06 | 3.61 | .04 | 3.59 | .03 | .42 | .655 |
| R superior frontal | 3.62 | .06 | 3.60 | .05 | 3.58 | .04 | .18 | .835 |
| L rostral middle frontal | 3.31 | .05 | 3.30 | .04 | 3.30 | .03 | .03 | .975 |
| R rostral middle frontal | 3.31 | .05 | 3.28 | .04 | 3.30 | .03 | .11 | .892 |
| L caudal middle frontal | 3.24 | .05 | 3.21 | .04 | 3.23 | .03 | .23 | .794 |
| R caudal middle frontal | 3.22 | .05 | 3.21 | .04 | 3.25 | .03 | .34 | .711 |
| L pars opercularis | 3.40 | .04 | 3.43 | .03 | 3.41 | .03 | .20 | .821 |
| R pars opercularis | 3.43 | .05 | 3.42 | .04 | 3.43 | .03 | .06 | .939 |
| L pars triangularis | 3.54 | .05 | 3.54 | .04 | 3.47 | .03 | 1.39 | .254 |
| R pars triangularis | 3.46 | .06 | 3.49 | .04 | 3.48 | .03 | .08 | .924 |
| L pars orbitalis | 3.77 | .06 | 3.72 | .05 | 3.80 | .04 | .85 | .430 |
| R pars orbitalis | 3.61 | .07 | 3.73 | .05 | 3.72 | .04 | 1.01 | .368 |
| L lateral orbital frontal | 3.72 | .05 | 3.66 | .04 | 3.70 | .03 | .66 | .520 |
| R lateral orbital frontal | 3.60 | .04 | 3.63 | .03 | 3.61 | .03 | .18 | .835 |
| L medial orbital frontal | 3.28 | .06 | 3.30 | .04 | 3.29 | .03 | .02 | .977 |
| R medial orbital frontal | 3.35 | .05 | 3.38 | .04 | 3.38 | .03 | .17 | .847 |
| L frontal pole | 3.69 | .11 | 3.87 | .09 | 3.82 | .07 | .76 | .473 |
| R frontal pole | 3.82 | .09 | 3.89 | .07 | 3.81 | .05 | .45 | .641 |
| L precentral | 3.26 | .05 | 3.26 | .04 | 3.23 | .03 | .23 | .793 |
| R precentral | 3.22 | .05 | 3.22 | .04 | 3.19 | .03 | .20 | .921 |
| L paracentral | 3.10 | .06 | 3.17 | .05 | 3.09 | .04 | 1.08 | .344 |
| R paracentral | 3.09 | .07 | 3.10 | .05 | 3.12 | .04 | .11 | .892 |
| **Parietal lobe** |  |  |  |  |  |  |  |  |
| L postcentral | 3.26 | .05 | 3.26 | .04 | 3.23 | .03 | .98 | .381 |
| R postcentral | 2.63 | .06 | 2.64 | .05 | 2.60 | .04 | .15 | .861 |
| L supramarginal | 3.28 | .05 | 3.28 | .04 | 3.27 | .03 | .02 | .979 |
| R supramarginal | 3.27 | .05 | 3.30 | .04 | 3.25 | .03 | .46 | .634 |
| L superior parietal | 2.79 | .06 | 2.80 | .05 | 2.75 | .04 | .35 | .705 |
| R superior parietal | 2.66 | .07 | 2.76 | .05 | 2.73 | .04 | .88 | .420 |
| L inferior parietal | 3.14 | .04 | 3.14 | .03 | 3.16 | .03 | .15 | .860 |
| R inferior parietal | 3.12 | .04 | 3.12 | .03 | 3.12 | .02 | .01 | .997 |
| L precuneus | 3.08 | .04 | 3.07 | .03 | 3.06 | .02 | .20 | .816 |
| R precuneus | 3.00 | .04 | 3.07 | .03 | 3.05 | .03 | .81 | .449 |
| **Temporal lobe** |  |  |  |  |  |  |  |  |
| L superior temporal | 3.48 | .05 | 3.51 | .04 | 3.46 | .03 | .60 | .552 |
| R superior temporal | 3.55 | .05 | 3.57 | .04 | 3.59 | .03 | .17 | .843 |
| L middle temporal | 3.62 | .04 | 3.65 | .03 | 3.57 | .02 | 2.20 | .116 |
| R middle temporal | 3.63 | .04 | 3.61 | .03 | 3.66 | .02 | 1.00 | .373 |
| L inferior temporal | 3.42 | .05 | 3.45 | .04 | 3.47 | .03 | .31 | .731 |
| R inferior temporal | 3.45 | .04 | 3.49 | .03 | 3.45 | .02 | .58 | .564 |
| L transverse temporal | 2.99 | .07 | 3.14 | .05 | 3.12 | .04 | 1.86 | .162 |
| R transverse temporal | 2.99 | .06 | 3.16 | .04 | 3.22 | .03 | 6.06 | .003** |
| L banks superior temporal sulcus | 3.14 | .05 | 3.16 | .04 | 3.10 | .03 | .70 | .498 |
| R banks superior temporal sulcus | 3.22 | .05 | 3.21 | .04 | 3.23 | .03 | .17 | .842 |

**Table S1** continued

| Regions | AH | | NH | | HC | | ANCOVA | |
| --- | --- | --- | --- | --- | --- | --- | --- | --- |
|  | Estimated marginal mean | SE | Estimated marginal mean | SE | Estimated marginal mean | SE | F | P |
| L entorhinal | 4.18 | .08 | 4.01 | .06 | 4.14 | .05 | 1.91 | .154 |
| R entorhinal | 4.25 | .08 | 4.23 | .06 | 4.29 | .05 | .32 | .726 |
| L parahippocampal | 3.34 | .09 | 3.30 | .07 | 3.16 | .06 | 2.08 | .130 |
| R parahippocampal | 3.27 | .07 | 3.25 | .05 | 3.23 | .04 | .12 | .885 |
| L temporal pole | 4.45 | .05 | 4.51 | .04 | 4.48 | .03 | .38 | .684 |
| R temporal pole | 4.54 | .05 | 4.63 | .04 | 4.54 | .03 | 1.74 | .181 |
| L fusiform | 3.40 | .04 | 3.37 | .03 | 3.35 | .02 | .62 | .538 |
| R fusiform | 3.34 | .03 | 3.34 | .03 | 3.33 | .02 | .01 | .990 |
| **Occipital** lobe |  |  |  |  |  |  |  |  |
| L lingual | 2.68 | .04 | 2.62 | .03 | 2.65 | .02 | .66 | .520 |
| R lingual | 2.72 | .03 | 2..71 | .02 | 2.72 | .02 | .05 | .948 |
| L pericalcarine | 2.32 | .06 | 2.35 | .04 | 2.28 | .03 | 1.00 | .372 |
| R pericalcarine | 2.35 | .05 | 2.32 | .04 | 2.33 | .03 | .13 | .880 |
| L cuneus | 2.57 | .04 | 2.60 | .03 | 2.57 | .03 | .27 | .761 |
| R cuneus | 2.59 | .05 | 2.59 | .03 | 2.60 | .03 | .03 | .968 |
| L lateral occipital | 2.97 | .04 | 2.93 | .03 | 2.94 | .03 | .32 | .726 |
| R lateral occipital | 2.94 | .04 | 2.98 | .03 | 2.99 | .03 | .48 | .619 |
| **Cingulate** cortex |  |  |  |  |  |  |  |  |
| L rostral anterior cingulate | 3.85 | .07 | 3.88 | .05 | 3.85 | .04 | .12 | .889 |
| R rostral anterior cingulate | 3.86 | .06 | 3.83 | .04 | 3.75 | .04 | 1.91 | .153 |
| L caudal anterior cingulate | 3.63 | .09 | 3.60 | .07 | 3.69 | .05 | .61 | .546 |
| R caudal anterior cingulate | 3.55 | .08 | 3.58 | .06 | 3.55 | .05 | .09 | .917 |
| L posterior cingulate | 3.39 | .03 | 3.46 | .04 | 3.45 | .03 | .61 | .547 |
| R posterior cingulate | 3.33 | .05 | 3.37 | .04 | 3.32 | .03 | .39 | .677 |
| L isthmus cingulate | 3.13 | .07 | 3.07 | .05 | 3.13 | .04 | .44 | .647 |
| R isthmus cingulate | 3.02 | .07 | 2.98 | .05 | 2.95 | .04 | .38 | .685 |
| **Insula cortex** |  |  |  |  |  |  |  |  |
| L insula | 3.90 | .04 | 3.87 | .03 | 3.87 | .02 | .22 | .804 |
| R insula | 3.97 | .04 | 3.89 | .03 | 3.93 | .02 | 1.35 | .265 |

** p< .01

HC: healthy controls; AH: hallucinating patients with schizophrenia: NH: non-hallucinating patients with schizophrenia.

^a^ Analyses of cortical thickness were performed in SPSS (http://www.spss.com) using general linear model, with age and sex as covariates.
